# Psychiatric disorders in teleoperators : A series of 27 cases

**DOI:** 10.1192/j.eurpsy.2023.1012

**Published:** 2023-07-19

**Authors:** A. Ghenim, D. Brahim, N. Mechergui, A. Belkahla, S. Ernez, G. Bahri, W. Ayed, I. Youssef, N. Ladhari

**Affiliations:** Occupational pathology and fitness for work, Charles Nicolle Hospital, Tunis, Tunisia

## Abstract

**Introduction:**

By its nature, the activity of teleoperators seems to be a propitious environment for the development of psychosocial disorders, sometimes severe

**Objectives:**

To study the frequency of psychiatric disorders among teleoperators and their impact on medical fitness for work

**Methods:**

This is a retrospective descriptive epidemiological study. We examined the files of teleoperators who were referred to the occupational medicine department of Charles Nicolle between 2014 and 2022 for a medical opinion of aptitude

**Results:**

A total of 82 cases were identified, 27 of which had psychological complaints. A clear female predominance was noted (21). The average age was 38.22+-6.536 years. All the patients were telephonists at the call-taking station with an average professional seniority of 9.3+-3.395 years. The symptoms noted were: sad mood (19), irritability (15), anxiety (10), sleep disorders (8), loss of vital impetus (8), neurovegetative signs (6), psychomotor slowing (5), concentration and memory disorders (3). Only one patient had suicidal thoughts. Symptoms had been evolving for an average of 34.32+-34.527 months. Psychiatric follow-up was noted in 16 patients. The diagnoses retained were: anxiety and depressive disorder (19), adjustment disorder (4), panic disorder (2), obsessive-compulsive disorder (1), and a satisfactory state of health (1). In some cases, the evolution was marked by complications: addiction(1), tonic stuttering with phobic disorder(1) and speech disorder(1). The prescribed treatments were: an antidepressant(2), an antidepressant-anxiety combination(8) and psychotropic drugs(2). Concerning the ability to work, 21 patients required an eviction from call taking (definitive (9) or temporary for 3 months (7) or 6 months (5) with re-evaluation of the medical ability to work at the end of this period), 1patient had an eviction from night work and an other had a reduction of the working hours.

**Conclusions:**

Teleoperators are exposed to several risks which can affect both their mental and physical health and put their medical fitness for work at risk.

**Disclosure of Interest:**

None Declared

